# Communication

**Published:** 1902-02

**Authors:** 


### COMMUNICATION.

The Secretary of the State Live-stock Sanitary Board has sent us the following curious communication. In case the suggestion proposed is not made use of by the Live-stock Sanitary Board of Pennsylvania, some of the other States of the Union may wish to avail themselves of this opportunity :

"——, JANUARY 23, 1902.

"TO THE STATE LIVE-STOCK BOARD, HARRISBURG, PA.

"I obtained Bulletin No 75 Tuberculosis of Cattle Pennsylvania for its Repression.

"I have a Plan to Stamp out the cause of the Tuberculosis. My Plan is no Fraud or quack I have experimented on My own Cattle and proved successful. I have allso in My Plan a preventive of epidemicks in Horses that I have used over 8 years and proved successful. I claim that Tuberculosis in Cows the Milk and butter or Meat or any product of Such Cows is dangerous to use as My plan will show. If the Live Stock Sanitary Board of Harrisburg, Pa will pay Me the compensation that I want I will forward the Plan to the Live Stock Sanitary Board of Harrisburg Pa. The compensation that I ask for My Plan is scarcely a handful according to the Money that has been spent and the loss to Stock owners not saying anything of the Human Lives that have been lost. I want Ten Thousand Dollars (10.000) All Nations should Appropriate towards the Stamping out of Tuberculosis. Now if the State Live Stock Sanitary Board of Harrisburg can obtain Fifty Thousand or one hundred Thousand I only want (10.000) Ten thousand and the Ballance the Four members can divide among themselves. If you do not understand Me come to see Me I dont practis veternary since the Law required a Veterinary to Regester.

"I did not Regester on account of ill health. My Plan Shall be writen to the point to the best of My knowledge and ability.

"Yours * * * *

"Gentle Men if I am not the proper Man to write and forward the plan in the Commonwealth of Pa throw this letter in the waste basket and inform Me who is a proper Man to write and forward the plan.

